# The application of covalent organic frameworks in Lithium-Sulfur batteries: A mini review for current research progress

**DOI:** 10.3389/fchem.2022.1055649

**Published:** 2022-10-21

**Authors:** Zhuo Wang, Fei Pan, Qi Zhao, Menglan Lv, Bin Zhang

**Affiliations:** ^1^ School of Chemistry and Chemical Engineering, Guizhou University, Guiyang, China; ^2^ School of Chemical Engineering, Guizhou Institute of Technology, Guiyang, China; ^3^ Yunnan Key Laboratory of Fungal Diversity and Green Development, Key Laboratory for Plant Diversity and Biogeography of East Asia, Kunming Institute of Botany, Chinese Academy of Sciences, Kunming, China

**Keywords:** covalent organic frameworks, lithium-sulfur batteries, cathode, anode, separator

## Abstract

Recently, how to enhance the energy density of rechargeable batteries dramatically is becoming a driving force in the field of energy storage research. Among the current energy storage technologies, the lithium-sulfur (Li-S) batteries are one of the most promising candidates for achieving high-capacity and commercial batteries. The theoretical energy density of Li-S batteries reaches to 2,600 Wh kg^−1^ with the theoretical capacity of 1,675 mA h g^−1^. Therefore, Li-S batteries are considered as the great potential for developing future energy storage technology. However, some of problems such as Li dendrites growth, the shuttle effect of sulfides and the electronic insulation feature of sulfur, have brought obstacles to the development of Li-S batteries. The covalent organic frameworks (COFs) are a series of porous materials with different topological structures, which show the versatile characteristics of high specific surface area, permanent pores, ordered porous channels and tunable internal structure. Potentially, their ordered channels and extended conjugated frameworks could facilitate rapid Li-ion diffusion and electron transport for the remarkable rate capability. On the basis of these merits, the COFs become the potential electrode materials to solve the above serious problems of Li-S batteries. In this mini review, we summarize the research progress of COFs utilized as electrode materials in the Li-S batteries, including the cathode, separator and anode materials. Accordingly, the outlook of COFs as electrodes for future development in Li-S batteries is also given.

## Introduction

Recently, the big problem of energy depletion seriously makes people attach importance to the development of clean and renewable energy to meet the increasing of energy demand (Shen, et al., 2021). Since 2021, the international crude oil price has continued to rise, while people’s demand for mobile power has been increasing gradually. In order to cope with this series of challenges, most countries have actively implemented energy-structure adjustment measures and strategies to lessen their reliance on fossil fuels and the greenhouse gas (GHG) emissions that result from their usage (Park, 2022; Bobba et al., 2020). So, the utilization and storage of new energy are the main strategies to replace fossil energy. Lithium-ion batteries (LIBs) are a promising option for electrical energy storage because of their high energy density and low cost, which has attracted wide attention (Brandt, 2018). Although LIBs dominate the consumption electronic market as the main commercial mobile power source, their energy density is approaching the limit and restricts the development of LIBs to a certain extent (Zhao, 2020). Lithium-sulfur (Li-S) batteries have a sulfur composite cathode, a polymer or liquid electrolyte, and a lithium anode, which are the promising candidate in the lithium battery series (Barghamadi et al., 2013). Li-S batteries have a shorter cycle life due to the natural insulating characteristics of sulfur and the shuttle effect of lithium polysulfides (LiPSs). Therefore, the commercial application of Li-S batteries continues to confront several obstacles, including side reactions, sulfur migration, and lithium dendrite development. (Xu et al., 2014).

## The bottleneck of Li-S batteries

The intrinsic electronic and ionic conductivity of sulfur is rather poor (Xu et al., 2018). Furthermore, the final products of the reaction in the charge-discharge process (Li_2_S_2_ and Li_2_S) are also electrically insulating, which is not conducive to for the batteries’ high rate performance (Li et al., 2021). Therefore, the electrons cannot be received by sulfur-based cathodes in the collector. Some researchers had tried to add conductive agents to cathode materials, but the theoretical capacity cannot be achieved due to the limited utilization of sulfur. In addition, in the course of a charge and discharge, the polysulfide intermediate produced from the positive electrode would dissolve into the electrolyte, which passes through the separator and diffuses to the negative electrode. This undesirable intermediate will react directly with the metal-lithium anode, where the negative effects of irreversible loss of active materials in the batteries, capacity fading, low efficiency and self-discharge are caused (Peng et al., 2017). Among this process, when charging and discharging, the fast volume change of sulfur and lithium sulfide will alter the morphology and structure of the positive electrode, resulting in the detachment of sulfur from the conductive framework and the ultimate decrease of capacity accordingly. Finally, the volume change of metallic lithium will cause the formation of dendrites in the lithium anode.

To solve these problems, scientists have carried out much studies on the Li-S battery materials. Among these fields, the emerging covalent organic frameworks (COFs) are widely used as electrode materials. COFs are a category of organic crystalline porous materials composed of light components including C, H, N, and O (Hu et al., 2018). They are two-dimensional (2D) or three-dimensional (3D) structural frameworks architected by light elements and strong covalent bonds, which are a type of pre-engineered polymer (Zeng et al., 2021). The COFs exist many advantages of low density, large specific surface area, adjustable pore size and structure, easy functionalization and plentiful combinations of covalent structures, in which they show great potentials in adsorbing polysulfides and inhibiting the formation of lithium dendrites.

## Research status of COFs in cathode materials

In recent years, because of the extensive research on COFs-based electrode materials in Li-S batteries, it can be seen that they are potentially great competitors for the high-performance cathode materials. Compared with other materials, COFs have a highly ordered nano-porous structure and a large specific surface area, where it is conducive to the immobilization of sulfur and to limit the loss of soluble polysulfides. In addition, ascribing to the feasible functionalization of COFs, the desirable skeleton structures of COFs with various pore sizes, shapes, and volumes can be flexibly designed at the molecular level.

**FIGURE 1:**
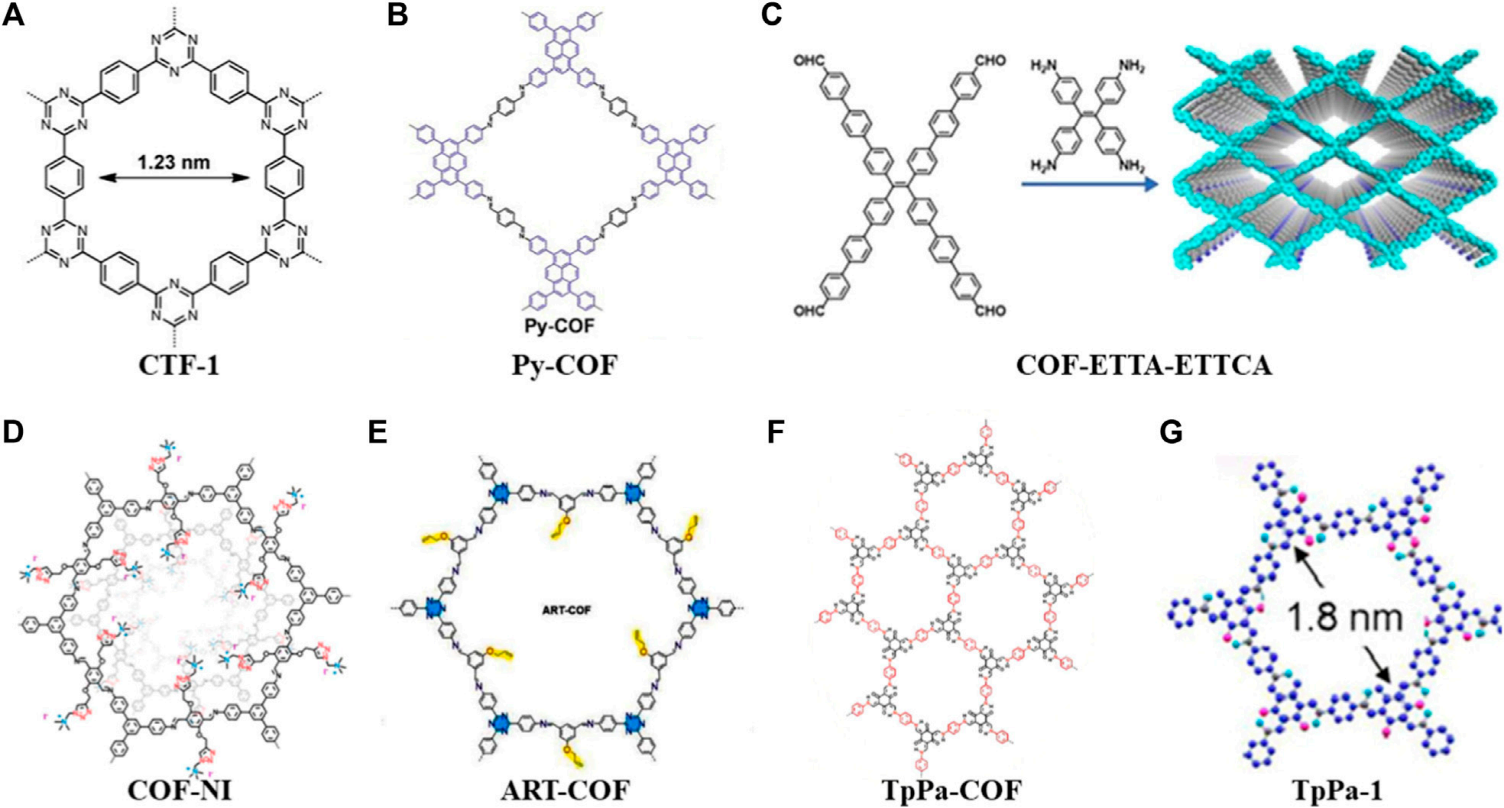
Summary of schematic diagram of the COFs in cathode materials **(A**–**G)** (Liao et al., 2014; Meng et al., 2018; Zhang et al., 2018; Lu et al., 2020; Wang et al., 2021; Li et al., 2022; Lin et al., 2022).

In 2014, Liao used a porous crystalline polymer backbone formed by the polymerization of benzene and triazine rings in a 2D structure (CTF-1) as a sulfur carrier material (Figure 1A). To achieve the composite cathode material, the CTF-1 and sulfur were mixed in a mass proportion of 3:2 at 155°C for 15 h. The thermogravimetric (TG) analysis showed that the loading of sulfur in the composite was about 34% (mass fraction). Subsequently, the electrochemical characterization showed that the nanopores of CTF-1 can effectively alleviate the dissolution and shuttling of polysulfides. After 50 cycles at a rate of 0.1°C, it still has 64% capacity retention rate. Accordingly, this work broadens the application of COFs in Li-S batteries (Liao et al., 2014).

However, the CTF/S cathode’s performance is clearly subpar. For instance, CTF-1 has a minor sulfur loading (34%). Two years later, A novel microporous COF (Por-COF) based on porphyrin was created by the solvothermal method. The composite material of Por-COF/S with a sulfur content of 55% was prepared in the melt-diffusion process, where it was used as a positive electrode material in Li-S batteries. At a rate of 0.5°C, the specific capacity remained at 633 mAh g^−1^ after 200 cycles, and the capacity decay rate per cycle was 0.16% (Liao et al., 2016). Then, they used 1,3,6,8-tetrakis (4-aminophenyl) pyrene (PyTTA) and terephthalaldehyde (TA) to synthesize Py-COF (Figure 1B) under the same method. After this composite cathode was cycled for 550 times at a rate of 5.0C, the capacity retention rate was 73.8% with a capacity decay rate per cycle of 0.048% (Meng et al., 2018).

In 2020, Lu et al. (2020) used π-conjugated system of aromatic tetraaldehyde (ETTCA) and tetrastyrene tetraamine (ETTA) to polycondensate under solvothermal conditions to gain a new COF material (COF-ETTA-ETTCA) (Figure 1C). The COF-ETTA-ETTCA was used for the loading of elemental sulfur directly, and the composite material (COF-ETTA-ETTCA-S) with a sulfur loading of 88.4% was achieved. It showed that the cathode material based on COF-ETTA-ETTCA-S exhibited a specific capacity of 1,617 mAh g^−1^ at 0.1°C, which was very nearly its predicted specific capacity. After 528 cycles at 0.5°C, it still maintained a high capacity with a capacity decay rate per cycle of 0.077%. These studies have proved that through COFs structure modification, the sulfur loading of active materials can be increased, and ultimately the electrochemical performance of Li-S batteries is improved simultaneously.

By introducing different functional groups in COFs, the composite cathode materials also present versatile electrochemical performance. Wang et al. (2021) grafted quaternary ammonium salt groups onto the pore channels of COFs in a one-pot procedure to create a cationic mesoporous COF (COF-NI) (Figure 1D). When cycled at 1.7–2.8 V, the electrode exhibits a high capacity of 1,038 mAh g^−1^ at a low cycling rate, and a high performance above 690 mAh g^−1^ is maintained even at 1°C or 2°C. This is attributed to the quaternary ammonium cations preventing the diffusion of polysulfide anions into the electrolyte through their strong interactions with polysulfide anions. Therefore, the functional groups enable the electrode to obtain good cycle performance and inhibit the shuttle effect. (Liao and Ye, 2018). Considering the strong affinity of allyl and triazine groups for lithium ions and the immobilization of sulfur by C-S covalent bonds, Li et al. (2022) reported a COF with allyl and triazine groups (ART-COF) (Figure 1E) to weaken the shuttle effect of LiPSs to the poles. This further provides ideas for developing batteries with high stability and long lifetime.

The low electrical conductivity of organic material composites is one of the shortcomings of current organic electrode materials, and some scientific research teams have developed strategies to solve this problem (An, et al., 2021). For instance, Zhang et al. (2018) prepared a novel core-shell covalent organic framework/multi-wall carbon nanotube nanocomposite (TpPa-COF@MWCNTs) by growing highly conductive multi-wall carbon nanotubes (MWCNTs) on porous TpPa-COF (Figure 1F). At a current density of 0.05 C, S/TpPa-COF@MWCNT-based cathode material showed a high initial discharge capacity of 1,242.2 mAh g^−1^. Its Coulombic efficiency was more than 99% when it was cycled at a current density of 0.5 C, and its ultralow capacity decay rate of 0.099% per cycle was observed after 450 cycles (Zhang et al., 2018). Furthermore, Lin et al. (2022) fabricated core-shell Co/Zr-NC@TpPa composites by coating a layer of TpPa-1 COFs (Figure 1G) on UIO-66-NH_2_-derived N-doped Co/Zr-NCs. The carbon matrix provides good electrical conductivity, while COFs immobilize sulfur and allow selective permeation of Li^+^. Furthermore, the doped Co and ZrO_2_ provide catalytic functions and trap active sites in Li-S batteries. (Lin et al., 2022).

In this section, the above discussion can summarize several ideas for COFs in solving the bottlenecks of Li-S batteries: 1) The physical sulfur fixation can be carried out by designing porous structures, and the nano-channels formed in COFs can effectively alleviate the dissolution of polysulfides and shuttle effect (CTF-1, Py-COF). 2) By expanding the conjugation system to obtain a high degree of conjugation and appropriate interlayer space, the loading of sulfur in the composite cathode can be increased dramatically (COF-ETTA-ETTCA). 3) The introduction of electron-rich heteroatoms or functional groups can fix polysulfides and reduce the loss of active substances (COF-NI, ART-COF). 4) Through mixing the highly conductive carbon-based materials with COFs or combining COFs with metal organic framework derived carbon/metal composites, the conductivity of cathode materials could be improved obviously (TpPa-COF, TpPa-1).

## Research status of COFs in anode materials

**FIGURE 2:**
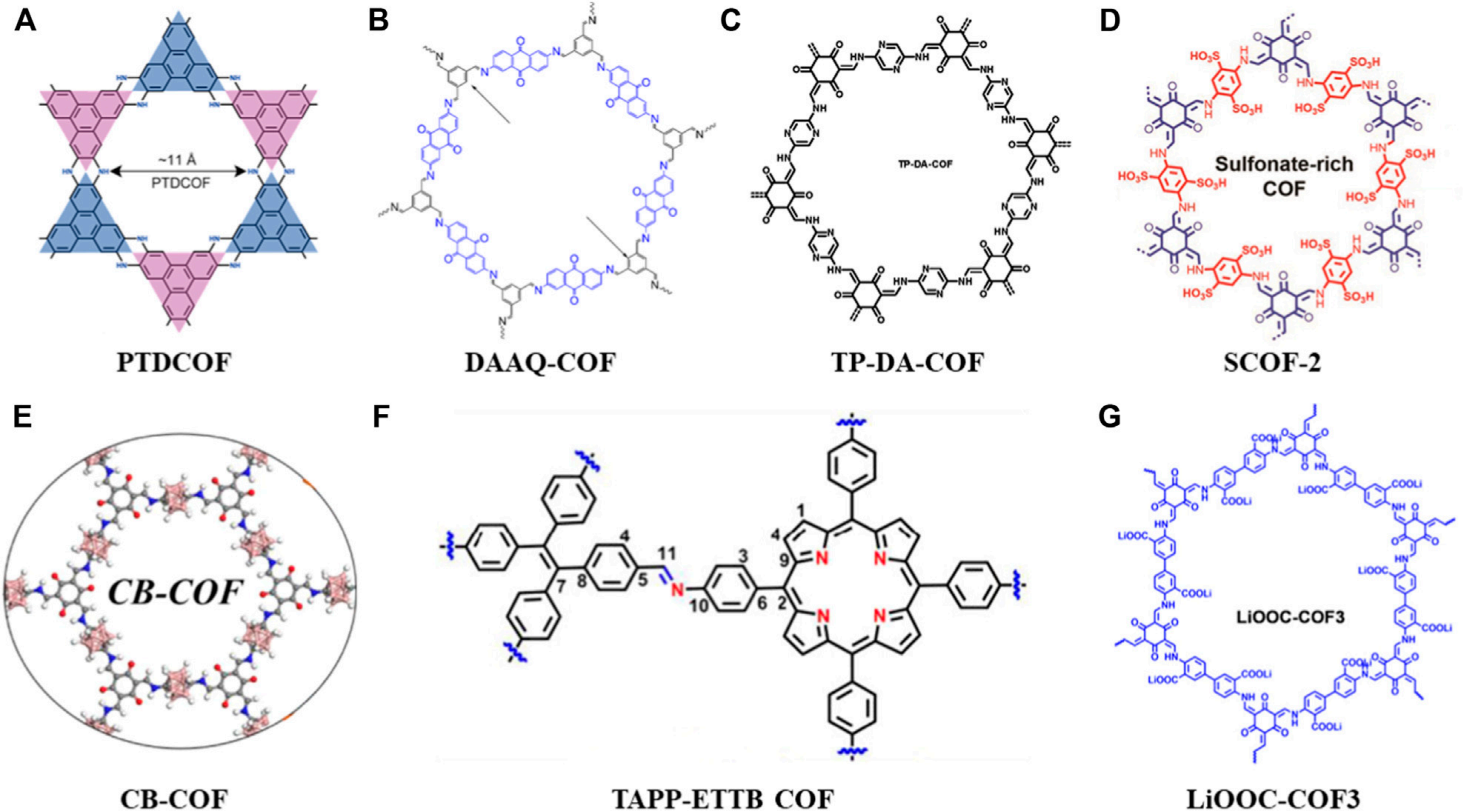
Summary of schematic diagram of the COFs in anode, separator and solid electrolytes materials **(A**–**G)** (Zhou et al., 2021; Zhao et al., 2021; Yang et al., 2022; Xu et al., 2021; Zhu et al., 2021; Sun et al., 2022; Zhao et al., 2022).

Since the anode is metallic lithium in Li-S batteries, the uneven deposition of lithium ions during charging tends to form dendrites, reaches the cathode by penetrating the separator. Eventually, an internal short circuit will lead to a severe safety danger (Wei et al., 2021). Therefore, fine design of a porous interface layer on the lithium anode can reduce the local current density and adjust the deposition while ensuring uniform Li^+^ flux, thereby improving the lithium loading capacity of the anode material. Zhou et al. (2021) designed and synthesized a piperazine-based two-dimensional COFs (PTDCOF) (Figure 2A). The PTDCOF was composed of benzo-acene units through irreversible piperazine bonds with regular pores and few layers. The composite PTDCOF had a capacity contribution of 1,644.3 mAh g^−1^ at 0.1 A g^−1^ with good rate and cycling performance.

The porous characteristics and high surface area of COFs make ions easily accessible to the redox active sites where they could improve the Li^+^ storage and diffusion. So, how to increase the number of redox active sites of COFs is crucial to meeting the high-capacity requirements of LIBs anodes (Zhai et al., 2022). Zhao et al. (2021) synthesized a COF material (DAAQ-COF) (Figure 2B) through the condensation of 2,6-diaminoanthraquinone (DAAQ) and 1,3,5-benzenetricarboxaldehyde (Tb). This is a layered porous COF with C=N and C=O dual redox active sites. The aromatic C=C bonds are also involved in the Li + storage mechanism (Zhao et al., 2021).

Yang et al. (2022) synthesized a novel redox COF (TP-DA-COF) (Figure 2C) *via in-situ* growing on CNTs with different COF thicknesses. The high capacity and efficient Li + diffusion in this composite was made possible by the large number of redox active sites that were exposed due to its few layers structure. More exposed active sites and better conductivity leaded to better electrical performance, where After 100 cycles at 0.1 A g^−1^ current density, the specific capacity was around 570 mAh g^−1^, and it stabilized at about 373 mAh g^−1^ with a Coulombic efficiency close to 100%.

In this section, the application of COFs in anode materials could solve the following problems: 1) By finely designing the porous interface layer, the local current density is reduced where the lithium ions can move to the redox active sites uniformly, thereby enhancing the lithium loading capacity of anode materials (PTDCOF). 2) To increase the number of redox active sites in COFs through designing layered porous COFs, it can increase the lithium loading capacity of anode materials (DAAQ-COF) 3) The COFs can also be uniformly encapsulated on other materials (e.g., carbon nanotubes) through *in-situ* reactions to obtain high crystallinity, ordered pores and conductive frameworks. Thus, more redox active sites are exposed and the distribution of lithium ions is adjusted to achieve dendrite-free lithium deposition (TP-DA-COF).

## Research status of COFs in separator materials

In the charge-discharge process, the positive electrode would generate a polysulfide intermediate (e.g., Li2Sx), which would then dissolve in the electrolyte, cross the separator, and diffuse toward the negative electrode. This polysulfide from the positive electrode could react directly with the metal lithium of the negative electrode, which eventually causes the irreversible loss of the effective material in the batteries. Since COFs have the potential to capture polysulfide anions due to their multi-pore structure and designable pore size, some works have proposed strategies to design functional separators to capture polysulfides.

Xu’s group designed and synthesized a dual-sulfonate COF (SCOF-2) (Figure 2D) to modify separators for Li-S batteries. When the SCOF-2 was combined with the concentrated sulfonic acid group, it would repel polysulfide anions and adsorb the molecular LiPS, where it acted as an ion sieve and promoted the migration of Li^+^ (Xu et al., 2021). More so than no/monosulfonate COFs, SCOF-2 hinders polysulfide migration and reduces lithium dendrite development due to its higher interlayer spacing and greater electron negativity. The high rate capacity of the SCOF-2 modified batteries was shown by electrochemical characterization to be 479 mAh g^−1^ at 5 C. Through the electrochemical characterization, results shown a high-rate capacity of 479 mAh g^−1^ at 5 C from the SCOF-2 upgraded batteries. After more than 800 cycles at 1 C, the decay rate was very low, at just 0.047% per cycle (Xu et al., 2021). With the same design concept, Zhu et al. (2021) synthesized a carborane-based amphiphilic COF (CB-COF) (Figure 2E) with the nano-trapping function of polysulfides. Its ability to synergistically trap LiPS and redeposit solid discharge products uniformly on the pore surface is remarkable, showing the potential to effectively suppress the shuttle effect. Furthermore, another strategy of inhibiting the polysulfides migration and reaction with lithium anode is to enhance the electron mobility for efficient electrocatalysis when effectively trapping LiPSs. For example, Sun et al. (2022) combined 5,10,15,20-tetrakis (4-aminophenyl) porphyrin (TAPP) and 4,4′,4″,4‴-(ethylene-1,1,2,2-tetra base) tetrabenzaldehyde, ETTB) through covalent network aggregation in a crystal framework to obtain TAPP-ETTB COF (Figure 2F). Then, through the introduction of graphene sheets to the COFs system, a TAPPETTB COF@G nanocomposite modified separator was obtained finally. This composite material simultaneously possessed a strong chemical affinity for LiPSs and excellent catalytic activity. It exhibited good cycling performance (920 mAh g^−1^ after 400 cycles at 0.2 A g^−1)^ and excellent rate capability (827.7 mA hg^−1^ at 2 A g^−1^) after the first cycle (Sun et al., 2022).

In this section, the COFs mainly solve two problems in the separators: 1) By introducing functional groups, it repels polysulfide anions and adsorbs molecular LiPS, thereby reducing the negative impact of the shuttle effect (SCOF-2, CB-COF). 2) Another strategy is to enhance the electron mobility for efficient electrocatalysis while trapping LiPS efficiently, providing sufficient lithiophilic sites for the strong chemisorption and catalysis of polysulfides (TAPP-ETTB COF).

## Research status of COFs in solid electrolyte materials

Until now, there is no very direct or strongly related research on COFs-based solid electrolyte in Li-S batteries (Gao et al., 2022). The chemical modification on COFs is beneficial to create excellent Li-ion conduction channels and improve the durability of LIBs (Huang et al., 2021). At present, some modified COFs have been developed as solid electrolytes for LIBs. For example, Zhao et al. (2022) constructed a lithium carboxylate COFs (LiOOC-COF3) (Figure 2G) with ordered one-dimensional channels. The single Li-ion conductor of LiOOC-COF3 exhibits an excellent ionic conductivity of 1.36 × 10^−5^ S cm^−1^ and a high mobility number of 0.91 at room temperature (Zhao et al., 2022), where the LIBs shows good cycling performance, high capacity output and no lithium dendrites. Therefore, the applicable potential of COF-related solid-state electrolyte in Li-S batteries can be expected in the future to construct ordered ion transport channels and guarantee the longevity and safety. However, the reaction mechanism between sulfur-based cathode and COF-based solid electrolyte is still unclear, so further research is needed to reveal it.

## Summaries and perspectives

This mini review mainly summarizes the research progress of COFs in cathode, anode, and separator materials in Li-S batteries. For the application of COFs materials in the cathode, researchers have improved the electrochemical performance of the batteries and suppressed the shuttle effect by constructing different pore environments and introducing different functional groups. Anode applications of COFs materials benefit from the incorporation of a porous interface layer that, when carefully designed, may effectively suppress the development of lithium dendrites. This is because it increases the lithium loading capacity of high anode materials. For COFs materials as separators, nano-collectors for polysulfides were designed, which can inhibit the shuttle of sulfides and also facilitate the conduction of lithium ions, thereby improving the electrochemical performance.

To develop high capacity and long lifetime Li-S batteries, the following research directions should attract more attention in the future: 1) The poor electrical conductivity of COFs materials makes it difficult to balance its cycle life and electrical conductivity, which is one of the main reasons that restrict its practical application regarding the technology of energy storage. 2) Due to the low volumetric energy density of COFs materials, the high specific capacity properties of cathodes composing of COFs materials have not been fully exploited. 3) Compared with the application of COFs around the design and development of cathode materials in Li-S batteries, their applications as separators, anode materials and solid electrolytes also show good prospects, which should be attracted much interests in further studies.
